# Supplementary material for: Effect of SARS-CoV-2 S protein on the proteolytic cleavage of the epithelial Na+ channel ENaC
Source: PLoS One. 2024 Apr 25;19(4):e0302436. doi: 10.1371/journal.pone.0302436 (PMC11045049; doi:10.1371/journal.pone.0302436)
Supplement: S1 File — (PDF) [file pone.0302436.s001.pdf]

SUPPORTING INFORMATION

Effect of SARS-CoV-2 S protein on the proteolytic cleavage of the Epithelial Na$^+$ Channel ENaC

Germán Ricardo Magaña-Ávila[a,b], Erika Moreno[a], Consuelo Plata[a], Héctor Carbajal-Contreras[a,c], Adrian Rafael Murillo de Ozores[a,b], Kevin García-Ávila[a], Norma Vázquez[d], Maria Syed[e], Jan Wysocki[e], Daniel Batlle[e], Gerardo Gamba[a,c,d] & María Castañeda-Bueno*[a]

Table of contents

Supplementary methods

Supplementary figure 1

Supplementary table 1

Supplementary figure 2

Supplementary figure 3

Supplementary figure 4

Supplementary references

Supplementary methods

Clones

Alpha, beta, and gamma ENaC constructs from rat in the pSD5 expression vector have been previously described and were obtained from Olivier Staub (1). A C-terminal HA tag was inserted in the αENaC cDNA and simultaneously subcloned into pGH19 by fast cloning (2). The original clone encoding the SARS-CoV-2 S protein was a gift from Dr. Nevan J. Krogan (3). The S protein cDNA was subcloned into the pGH19 expression vector and FLAG- tagged after a linker region (PPVAT) in the C-terminus (4). The S protein clone harbouring the deletion of the multibasic furin cleavage site was generated by fast cloning (2).

Immunoblots

Forty-eight hours after injection, oocytes were lysed mechanically by pipetting, with 5 ul per oocyte of lysis buffer (50 mM Tris-HCl (pH 7.5), 1mM EGTA, 1 mM EDTA, 50 mM sodium fluoride, 5 mM sodium pyrophosphate, 1 mM sodium orthovanadate, 1% (wt/vol) Nonidet P-40 and 0.27 M sucrose, protease inhibitors (Complete, Roche)). Lysates were centrifuged at 13,000 rpm for 10 minutes at 4°C and supernatants were recovered. Protein concentration was quantified by the BCA protein assay. Lysates were separated by SDS-PAGE on 7.5% gels. Proteins were electrotransferred onto PVDF membranes and immunoblotting was performed. Membranes were blocked for 1 hour in 10% (wt/vol) non-fat milk (BioRad) dissolved in Tris-buffered saline containing 0.1% Tween20 (TBSt). Incubation with primary antibodies was conducted overnight at 4°C and with secondary antibodies for 1 hour at room temperature. The following primary commercial HRP-conjugated antibodies were used: anti-Flag 1:5000 (Sigma-Aldrich, A8592), anti-HA 1:1000 (SigmaAldrich, H6533), anti-beta-actin 1:10000 (Santa Cruz Biotechnology, sc-47778). All antibodies were diluted in 5% (wt/vol) non-fat milk (BioRad) dissolved in TBSt. Bound antibodies were detected by chemiluminescence using the Luminata Forte Western HRP substrate (Millipore). For the densitometric analysis of samples across different gels, densitometries of each individual gel were first quantified, and the values of each sample were

normalized to the average intensity of the samples within the gel. For the analysis of ENaC processing, values from the cleaved band (~65 kDa) were divided by the value of the uncleaved band (~90 kDa). The graph displays values relative to the control (ENaC without SPIKE). Group comparisons were performed using analysis of variance (ANOVA), followed by Tukey post hoc test.

Supplementary Figure 1. Representative example of traces of whole-cell currents recorded at -140 mV by two-voltage clamp of *Xenopus laevis* oocytes injected with water (A), ENaC cRNA (α,β, and γ subunits) (B), ENaC + S^{WT} cRNAs (C), or ENaC + $S^{\Delta PRRA}$ cRNAs (D) in the absence and presence of 10 µM amiloride. The experiments presented are from the same batch of oocytes and were done on the same day. The panels to the right show I/V plots of for oocytes injected with water (E), ENaC cRNA (α,β, and γ subunits) (F), ENaC + S^{WT} cRNAs (G), or ENaC + $S^{\Delta PRRA}$ cRNAs (H) in the absence and presence of 10 µM amiloride. Data recorded in three independent experiments was used to produce these plots.

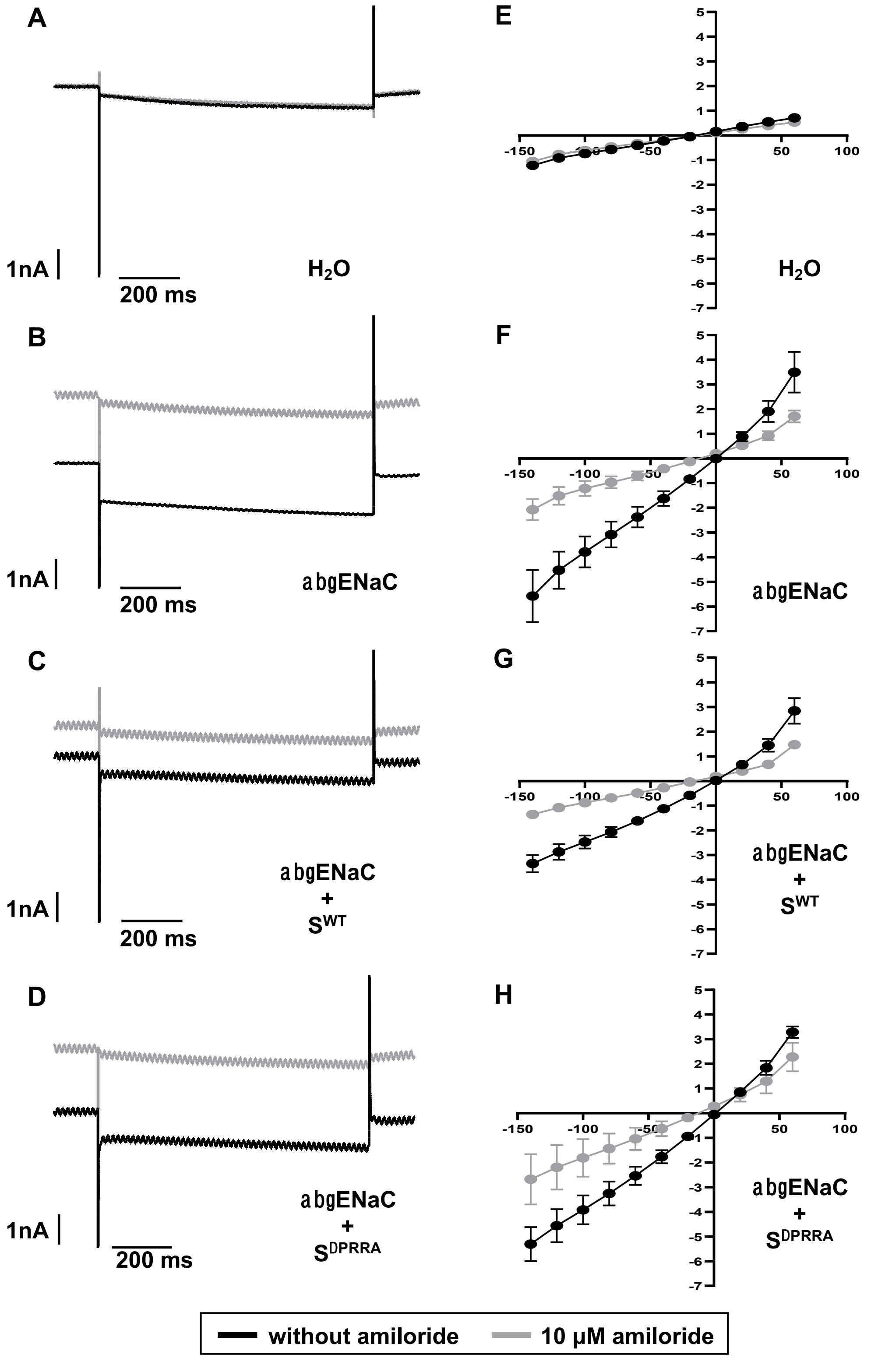

Supplementary Table 1. Two-way ANOVA of voltage-current data analysis. Using Prism (GraphPad Software) we performed a Two-way ANOVA and a post-hoc Tukey test to evaluate the voltage-current curves from the different experimental groups. Statistical significance was considered using a p value <0.05

ANOVA table	SS	DF	MS	F (DFn, DFd)	P value
**Interaction**	7.127	20	0.3563	F (20, 66) = 1.047	P=0.4242
**Row Factor**	162.6	10	16.26	F (10, 66) = 47.76	P<0.0001
**Column Factor**	3.959	2	1.98	F (2, 66) = 5.816	P=0.0047
**Residual**	22.47	66	0.3404		

Tukey's multiple comparisons test	Mean Diff.	95.00% CI of diff.	Significant?	Summary	Adjusted P Value
**ENaC vs. ENaC-S^{wt}**	-0.4671	-0.8115 to -0.1227	Yes	**	0.0051
**ENaC vs. ENaC-$S^{\Delta PRRA}$**	-0.1058	-0.4502 to 0.2386	No	ns	0.7426
**ENaC-S^{wt} vs. ENaC-$S^{\Delta PRRA}$**	0.3613	0.01691 to 0.7057	Yes	*	0.0376

Supplementary figure 2. Validation of γENaC antibody in kidney tissue. A) Immunoblots were performed with kidney protein extracts obtained from wild type mice treated with vehicle or amiloride (6 μg/g/day for 4 days). Amiloride is an inhibitor of ENaC that was used at a high dose that causes hyperkalemia and volume depletion. Under these conditions ENaC cleavage is expected to increase. B) Immunofluorescent staining of slices of kidney tissue obtained from wild type mice treated with vehicle or amiloride. Antibodies against γENaC and B1 ATPAse were used. The expected signal was observed in principal cells (for γENaC) and intercallated cells (for B1 ATPase). ENaC relocalization to the apical membrane was observed in amiloride-treated mice. 100 μm scale bars are shown.

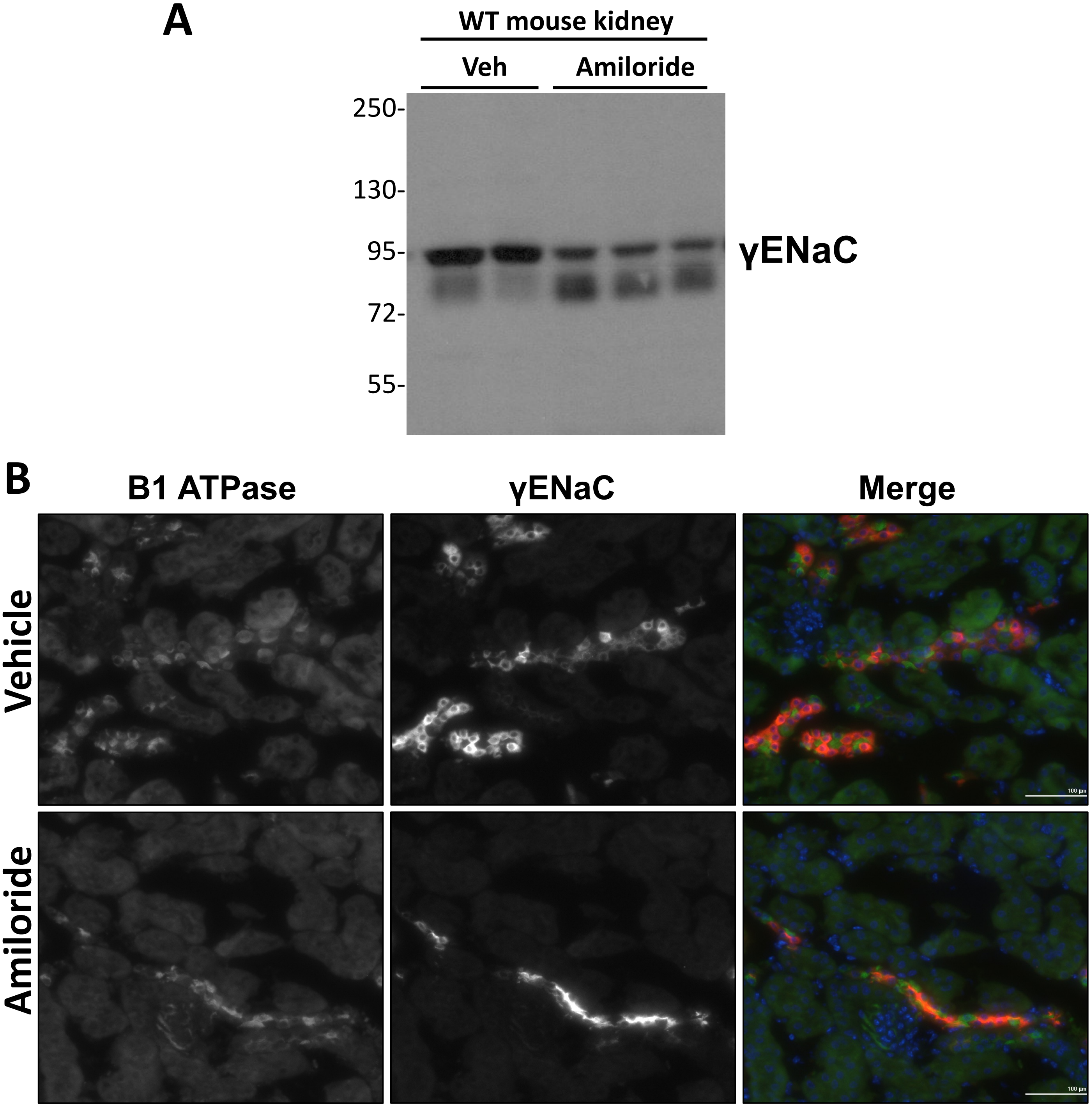

Supplementary figure 3. Validation of γENaC antibody in lung tissue. Immunofluorescent staining was performed with antibody against γENaC in slices from kidney and lung tissue of uninfected wild type mice (A) and SARS-CoV2 infected mice K18 mice (B). Signals of higher and lower intensity were observed in lung tissue. This pattern was similar to that observed with an antibody against the Surfactant protein A (a marker of type 2 pneumocytes, where ENaC is known to be expressed)(see Figure 3 of manuscript). Negative control was done by omission of primary gENaC antibody (B, right panel).

A WT mice (uninfected):

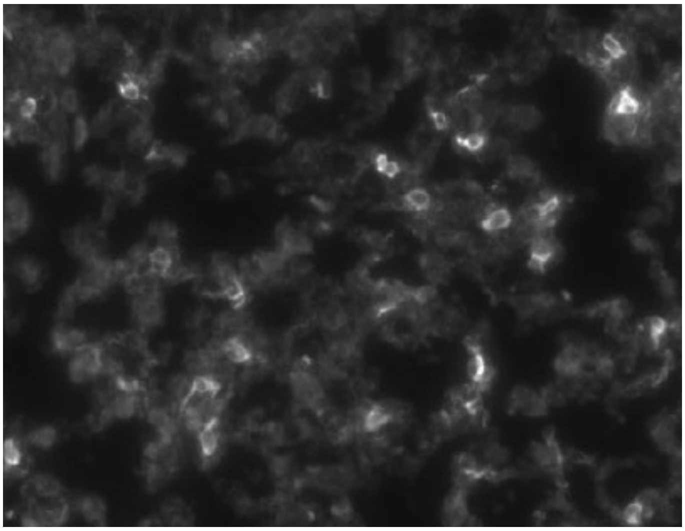

B K18 infected mice:

Kidney | **Lung** | **Lung No primary antibody**

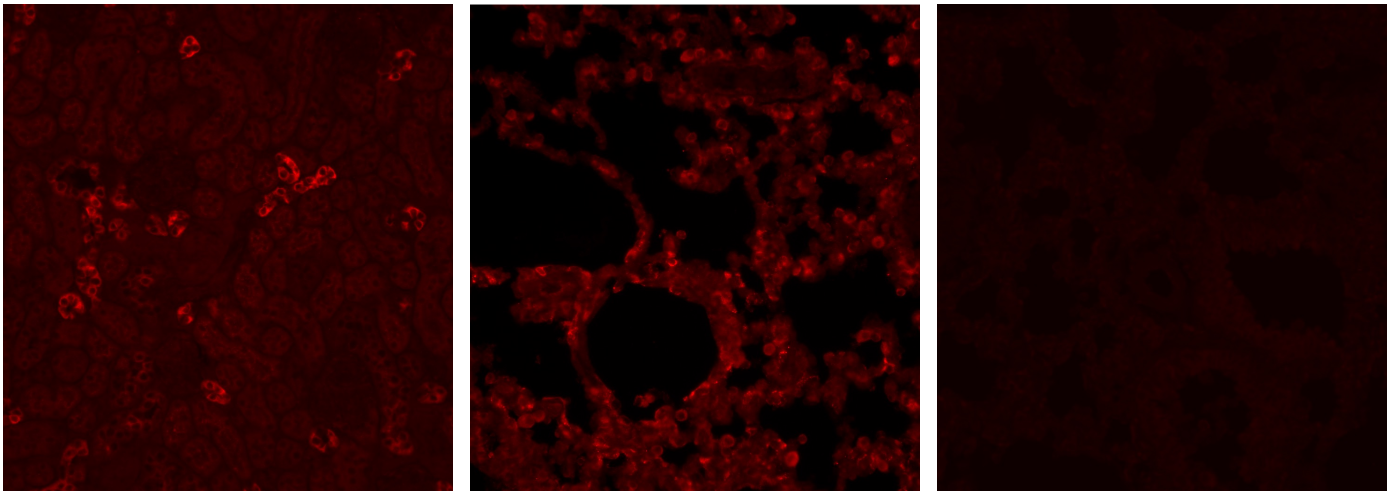

Supplementary figure 4. Immunofluorescence staining for γENaC (red) in lung sections of three uninfected control WT C57Bl6 mice (panels A-C) compared to three k18hACE2 transgenic mice infected with SARS-CoV-2 (panels D-F). SARS-CoV-2 Nucleoprotein staining (green) in the same three infected mice (G-I). White arrows point to areas of staining. No clear differences are apparent between control and infected mice. All pictures taken at 40x magnification.

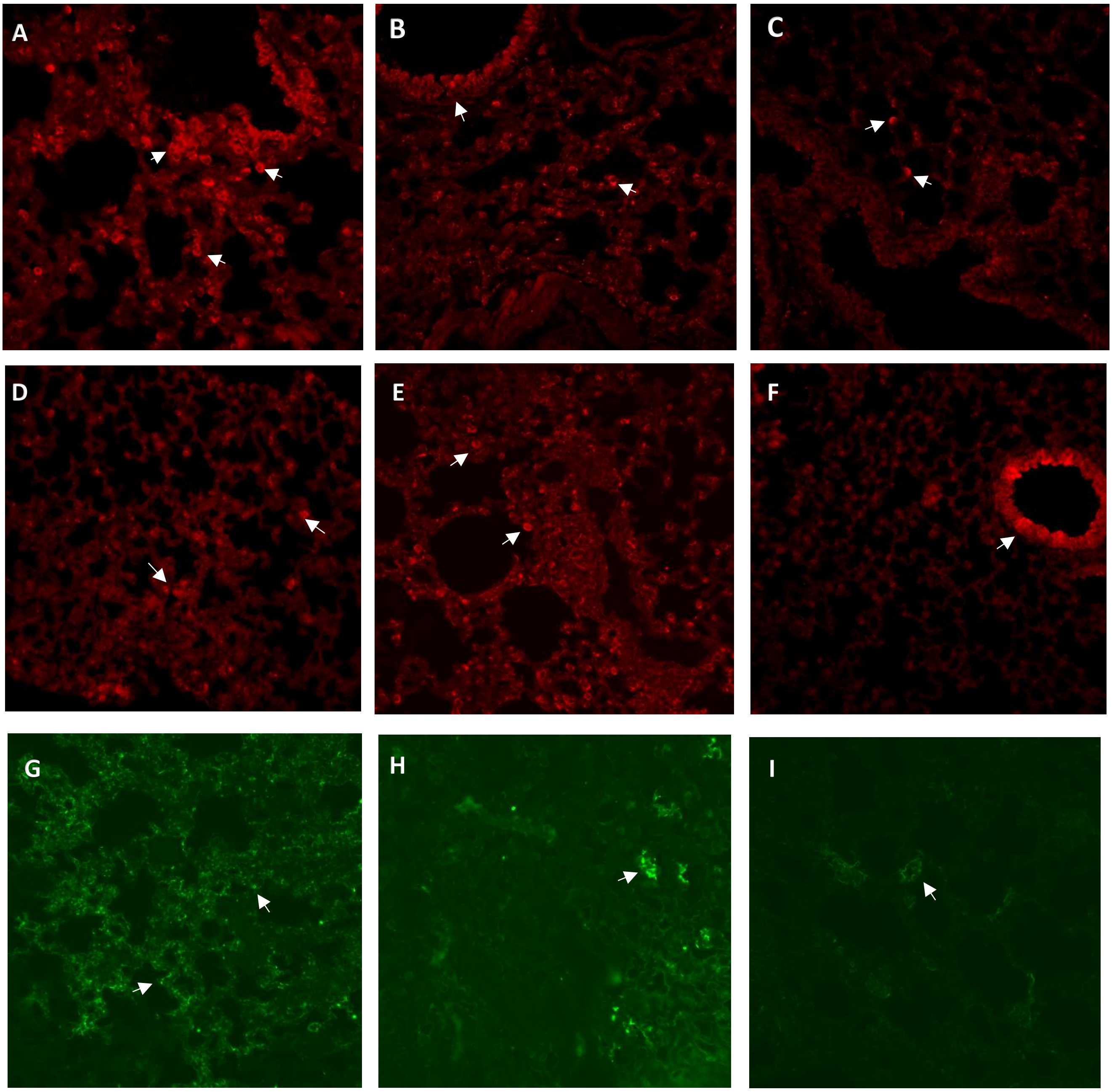

Supplementary references

1. Staub O, Gautschi I, Ishikawa T, Breitschopf K, Ciechanover A, Schild L, et al. Regulation of stability and function of the epithelial Na+ channel (ENaC) by ubiquitination. EMBO J. 1997;16(21):6325–36.
2. Li C, Wen A, Shen B, Lu J, Huang Y, Chang Y. FastCloning: a highly simplified, purification-free, sequence- and ligation-independent PCR cloning method. BMC Biotechnol [Internet]. 2011;11(1):92. Available from: http://bmcbiotechnol.biomedcentral.com/articles/10.1186/1472-6750-11-92
3. Gordon DE, Jang GM, Bouhaddou M, Xu J, Obernier K, White KM, et al. A SARS-CoV-2 protein interaction map reveals targets for drug repurposing. Nature. 2020;583(7816):459–68.
4. Bosch BJ, de Haan CAM, Rottier PJM. Coronavirus Spike Glycoprotein, Extended at the Carboxy Terminus with Green Fluorescent Protein, Is Assembly Competent. J Virol. 2004;78(14):7369–78.
