# Supplementary material for: Effect of SARS-CoV-2 S protein on the proteolytic cleavage of the epithelial Na+ channel ENaC
Source: PLoS One. 2024 Apr 25;19(4):e0302436. doi: 10.1371/journal.pone.0302436 (PMC11045049; doi:10.1371/journal.pone.0302436)
Supplement: Attachment — Submitted filename: Response to reviewers.pdf [file pone.0302436.s002.pdf]

RESPONSE TO REVIEWERS

Reviewer #1: In this manuscript by Magaña-Avila et al., the authors investigate whether a competition exists between SARS-CoV2 Spike protein and the alpha subunit of ENaC for cleavage by furin, using a Xenopus laevis coexpression system in vitro (WB and patch clamp) and ENaC-gamma subunit expression in immunofluorescence of lungs from SARS-CoV2-infected K18hACE2 mice. The authors conclude that a bi-directional competition exists for furin cleavage between S protein and ENaC-alpha and that this represents a main mechanism for SARS-CoV2-induced ENaC dysfunction.

Major comments.

1. Data presentation is very poor, in view of a complete lack of detail and of essential controls. As such, Western blots do not include MW markers or loading controls. The immunofluorescence images lack antibody isotype controls and quantification.

R: We are sorry that the reviewer got this impression. We have now modified the manuscript in an attempt to improve the reviewer´s perception of the manuscript. We have included loading controls and additional MW markers to those originally included.

We have also included as supplementary information some validation data for the gamma ENaC antibody used for immunofluorescence (Supplementary figures 2 and 3). In Western blot with kidney samples we were able to show that the antibody recognizes only two bands of the expected molecular weights for the uncleaved and cleaved forms of γENaC (Figure S2). Importantly, the expected increase in the cleaved form of γENaC was observed in samples from animals treated with amiloride, which develop severe volume depletion and hyperkalemia, and thus, in which ENaC activation and γENaC cleavage is expected. This shows that the antibody is able to specifically recognize γENaC in WBs with kidney tissue samples.

In addition, in immunofluorescent staining of kidney samples, strong tubular staining with very little background fluorescence was observed (Figure S2). Signal was localized to principal cells of the nephron that were negative for ATPase B1 staining, which marks intercalated cells in connecting tubules and collecting ducts.

In lung tissue (Figure 3 and Figure S3), signals of higher and lower intensity were observed. This pattern was similar to that observed with an antibody against the Surfactant protein A (a marker of type 2 pneumocytes, where ENaC is known to be expressed) (Figure 3). Similar staining was observed in lungs from non-infected WT mice and K18 infected mice. Negative control was done by omission of primary γENaC antibody in the IF procedure that was substituted by incubation with the diluent only (Figure S3).

Finally, it is worth mentioning that the used γENaC antibody has been previously validated by others (1).

2. The conclusion of the authors is not supported by the data provided, rather the opposite is true. An S protein mutant with a deficient furin cleavage site inhibits ENaC-alpha cleavage (MW of band not described!) nearly with the same efficacy as wt S protein (Fig. 1). The described inhibition of S protein cleavage by the presence of ENaC-alpha is not clear and the lane numbers are not indicated.

R: It is true that, as the reviewer states, the S protein mutant with a deficient furin cleavage site still appears to significantly prevent αENaC cleavage. It is only at the lowest S protein concentration tested (0.05 ug/ul)

that a difference between the wild type S protein and the mutant S protein is appreciated. In the discussion, we speculate that perhaps the mutant S protein may still be able to bind with slightly less affinity to the protease and in this way exert a competitive effect that prevents ENaC cleavage. We cannot rule out however, that other explanations may exist. For instance, the S protein may inhibit ENaC cleavage by another mechanism other than competition. However, we think that the observation that ENaC´s presence also prevents S protein cleavage supports the idea of competition. Interestingly, supporting the idea that the S protein affects an early step in the channel's processing and /or trafficking to the membrane, it has been shown in oocytes that injecting the mRNA encoding for the S protein 24 h after injection of ENaC mRNA does not result in inhibition of ENaC activity (2). This evidence has now been cited in the manuscript and the above discussion has now been included.

We think that our data does indeed show clear inhibition of S protein cleavage by ENaC co-expression. We emphasize this in the legend of figure 1: "It is noteworthy that proteolytic processing of S^{WT} was clearly prevented in the presence of ENaC coexpression (bands corresponding to the cleaved S2 subunit observed in oocytes injected with the same amount of S^{WT} in the absence or presence of ENaC are highlighted with boxes)." We also emphasize this in the results section: "The proteolytic processing of S^{WT} was also appreciated in the immunoblots. As expected, this processing was not observed for the $S^{\Delta PRRA}$ mutant. It is noteworthy that in the presence of ENaC, S^{WT} processing was reduced (compare **lane 2 with lane 7** of the blot in Fig.1 in which equivalent levels of S^{WT} cRNA were injected)." We have now included the lane numbers in Figure 1 to facilitate identification of the lanes mentioned in the text.

In addition, we now include new blots (figure 1C-D) from experiments in which the amount of S protein was kept constant and different combinations of ENaC subunits in different amounts were co-expressed. In these experiments, we confirmed that, as previously shown by others (3), it is necessary for the three ENaC subunits to be present to observe αENaC and γENaC cleavage. Moreover, we observed that only in the presence of the three subunits, S protein cleavage decreases and that as gENaC concentration increases and more cleavage of γENAC and αENaC is observed, less cleavage of the S protein is observed.

3. Patch clamp data, which should include an amiloride control, only show a minor decrease in ENaC currents due to co-expression with S protein and only for certain voltages. Here mutant S protein does not affect the currents although in Fig 1 its co-expression clearly inhibits ENaC-alpha cleavage. As such, there is a complete disconnect between data in Figs 1 and 2.

R: We apologize for the confusion. We would like to clarify that the two-electrode voltage clamp results reported already consider the amiloride control. This is, for every given voltage tested, the current values plotted are the values obtained in the absence of amiloride minus the values obtained in its presence. The current-voltage relationship of amiloride-sensitive currents that we observed is similar to that reported by others (4). Please refer to the "Two-electron voltage clamp" section in the Materials and Methods section where a description of these calculations is included.

This comment by the reviewer actually motivated us to reanalyze our electrophysiological data. In our new analysis we now consider the average current from all oocytes in each experiment instead of considering current values from individual oocytes. We decided to do this to balance the weight given to each experiment, given that different amounts of oocytes were studied in each experiment. To evaluate if there were differences between the curves, we performed a two-way ANOVA of the repeated samples and a post-hoc Tukey test showing significative statistical difference between the ENaC and ENaC + S^{WT} groups.

As the reviewer points out, only a small but significant decrease in ENaC-mediated currents was observed in the presence of S protein. This is despite the apparently complete prevention of αENaC cleavage shown in the blots represented in figure 1. We do not believe, however, that this is unexpected, as others have reported only partial decrease in ENaC currents with mutant channels in which the cleavage site is mutated and thus cleavage is completely prevented (3).

Finally, it is true that we also expected to see a decrease in ENaC currents in the presence of the mutant S protein, given that this mutant also significantly prevents ENaC cleavage. We do see a tendency for the ENaC currents to be smaller in the presence of the S mutant. However, these are not statistically significant due to the data variation. Interestingly, however, others have also reported that ENaC-mediated currents decrease in the presence of the wild type S protein and that the inhibition observed is only partially prevented with a mutant S protein (2). This work is now cited in our manuscript (page 10).

4. The co-expression system is artificial since it ignores the signal transduction initiated by binding of S protein to ACE2 in the lungs. ACE2 downregulation upon S protein binding impairs the ACE/ACE2 balance and can activate PKC, a known inhibitor of ENaC open probability (Bao et al., Am J Physiol Renal Physiol. 2007; Chen et al., Am J Physiol Lung Cell Mol Physiol. 2004).

R: We appreciate this comment that has given us the opportunity to discuss the possible role of PKC and other proposed mechanisms that may contribute to ENaC disfunction in our manuscript (see discussion section). Fortunately, the role of PKC has been previously addressed by others and Grant et. al. have shown that inhibition of PKC with Go¨-6976 do not prevent the decrease in ENaC-mediated Na+ currents in oocytes co-injected with SARS-CoV-2 S protein (2). It has also been shown that treatment with the recombinant Receptor Binding Domain of the S protein can induce ENaC inhibition in human lung microvascular endothelial cells and this has been proposed to contribute to lung vasculature disfunction (5). This inhibition correlates with a reduction in ACE2 surface expression and generation of reactive oxygen species. And given that only extracellular exposure to the Receptor Binding Domain of the S protein is necessary to induce ENaC inhibition, the mechanism of inhibition is probably unrelated to an effect on ENaC cleavage. These works are now cited in the manuscript.

We agree that in our system we may have missed other possible mechanisms for ENaC inhibition by SARS-CoV2. Nevertheless, we do not attempt to rule out other possibly involved mechanisms, rather to contribute by addressing this particular mechanism that involves defective ENaC cleavage and for which the oocyte system appears to be suitable.

Reviewer #2: In their study, Magana-Avila et al. report the competitive inhibition of cleavage of the alpha subunit of the epithelial sodiun channel ENaC by coexpression of SARS-CoV 2 spike protein in the Xenopus oocyte expression system. Moreover, the authors find that this coincides with reduced ENaC activity in TEVC experiments. Unfortunately, the authors could not present evidence for the impact in vivo since lungs from infected mice were not available for Western blot.
The study is straightforward and the results are presented in a clear and understandable fashion. I particularly like Fig 4 generating a hypothesis for the pathophysiological impact of the findings.
I have the following suggestions:

1. Fig 1: why is there inhibition of aENaC cleavage at 0.05 spike protein expression whereas cleavage of spike protein is only detectable at 0.4 spike protein expression?

R: This is probably a misunderstanding. Cleavage of spike is detected at lower levels of spike expression. For instance, in lane number 2 of the blot, it can be seen that when spike is injected at 0.2 ug/ul cleavage is observed. However, with this same amount of spike, but in the presence of ENaC (lane #7) no cleaved spike is observed probably due to competition with ENaC.

2. Fig 1: It would be interesting to see data on cleavage of gENaC as well since furin is supposed to cleave gENaC once near the N terminus. I suggest the authors to present data on the cleavage of gENaC using the same antibody as used for IF (Stressmarq SPC-405)?

R: We have performed blots of gamma ENaC as suggested by the reviewer. Interestingly, in these blots we observed that γENaC appears to be more efficiently cleaved than αENaC because the proportion of cleaved/full length γENaC is higher than that observed for αENaC and spike (see for example figure 1C, lane 5, in which equal amounts of αENAC and γENaC were injected). We do not see, however, a clear negative effect of spike expression on γENaC cleavage as that observed for αENaC. This effect was only observed in some blots and at high levels of spike expression. Below you can see representative blots for both situations (cleavage prevented and not prevented). We believe this could be due to the higher efficiency of cleavage of γENaC. This is, if γENaC is preferentially cleaved, then perhaps it is more difficult to observe prevention of cleavage by spike´s presence. Alternatively, cleavage of γENaC by another protease could also explain these observations. These observations, however, do not rule out that gamma ENaC cleavage could be affected in vivo in infected mice under conditions in which S protein expression may be much higher than ENaC expression.

Our observations suggest that it might be impossible to dissect if it is the presence of αENaC, γENaC, or both what prevents spike cleavage. In the new figure 1C-D we show that only in the presence of the three subunits, alfa and gamma ENaC are cleaved (this has also been shown by others (3)). Of note, it is also only in the presence of the 3 subunits of ENaC that the cleavage of spike is inhibited. In other words, only when cleavage of ENaC is possible, the inhibition of the cleavage of spike is observed, and as γENaC concentration increases and more cleavage of γENAC and αENaC is observed, less cleavage of the S protein is observed.

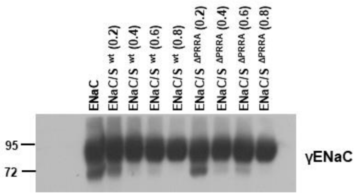

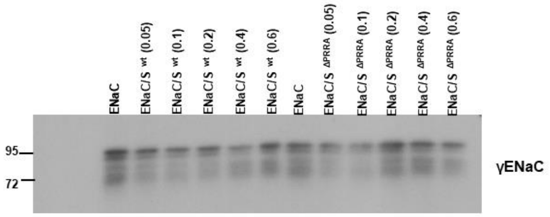

3. Fig. 1: please indicate molecular size of cleaved aENaC.
This information has been included in the figure legend.

4. Fig 2: please give sample traces of amiloride-sensitive currents for all groups at e.g. -140 mV holding potential

R: Traces have been included in supplementary figure 1.

5. Fig 3: the background seems to high. Can the authors optimize the conditions by diluting the antibody or improving antigen retrieval? The authors should also give a view with a higher magnification as inset.

R: Thank you for this comment. As described in response to comment 1 of the Reviewer 1, we did a careful validation of the γENaC antibody that was used for immunofluorescence (IF) (Figures S3 and S4). This validation suggests that the observed signal in IFs of lung tissue is specific. We tested different antigen retrieval protocols and also IF with no antigen retrieval procedure. For the images presented in figures 3, S2 and S3, we used the protocol in which best results were obtained with kidney and lung samples. As mentioned before, to rule out non-specific staining in the lungs, negative control of lung tissue with omission of primary antibody was performed which resulted in virtually no fluorescence. This suggested that the IF signals of lower intensity observed in the lung tissue are likely not background related.

As the reviewer suggested, we now provide insets of higher resolution in the new Figure 3.

Reviewer #3: I have reviewed the manuscript Effect of SARS-CoV-2 S protein on the proteolytic cleavage of the Epithelial Na+ Channel ENaC by Bueno et al.
Authors hypothesize that the S-protein competes for furin clevage with the alpha (and gamma) ENaC subunit in lung and that this could contribute to inactivate ENaC and promote fluid accumulation. The hypothesis is tested in vitro in the Xenopus expression system and in vivo in mice exposed to SARS Cov-2 virus. Oocytes were injected with mRNA for all EnaC subunits and with Sprotein.
Alpha ENaC cleavage and inward amiloride-sensitive currents were attenuated by S-protein overexpression but not in S-protein with mutated furin-cleavage site. Only immunofluorescence was possible in infected mouse lungs. Data indicate that overexpression of S-protein engages furin and leaves alpha ENaC uncleaved.
The idea is good and novel and well presented in the introduction. I have some suggestions that authors could consider in order to improve their manuscript.

1. From which species were the mRNAs that were injected?

R: Thanks for making us note that we missed this information. The mRNAs of ENaC subunits injected were from rat. We have now included this information in the "Materials and Methods" section.

2. Why was furin not overexpressed and where does the proposed reaction occur? In the biosynthesis pathway or on the surface? I realize there is a schematic drawing but no data are presented on this issue.

R: We did not think necessary to overexpress furin, as it has been shown by others that furin-mediated cleavage can occur in this oocyte system (3, 6, 7). Furin is a serine protease member of the proprotein convertase family that resides primarily within the trans-Golgi (TGN) network but also cycles to the cell surface (8, 9). It is thus generally assumed that ENaC processing by furin takes place during its trafficking through the TGN to the plasma membrane (10). Therefore, what we propose is that during the synthesis of the SARS-CoV2 S protein and its passage through the TGN the competition with the ENaC subunits takes place.

Supporting the idea that the S protein affects an early step in the channel's processing and /or trafficking to the membrane, there is published evidence from experiments performed in oocytes showing that injecting the mRNA encoding for the S protein 24 h after injection of ENaC mRNA does not result in inhibition of ENaC activity (2). This evidence has now been cited in the manuscript (Discussion section).

3. Is the amount of furin always the same or in other words, does the alleged competition not also depend on the amount of furin?

R: We assume that the amount of furin may vary between batches of oocytes obtained from different frogs. However, since we overexpressed the ENaC and spike proteins in our system, we expected these proteins to be in excess with respect to furin. Thus, under these conditions we expected that competition would be more easily observed.

4. Can it be excluded that other inherent/endogenous proteases contribe to cleavage?

R: No this cannot be excluded. In the literature it is generally assumed that furin participates in ENaC proteolytic processing because there is evidence that this occurs (6, 7). Particularly, furin has been involved in cleavage at two positions in αENaC and one position in γENaC. However, it cannot be excluded that other furin-like proteases may participate (11, 12). We have now modified the text of the manuscript to mention cleavage by "furin-like proteases" instead of just "furin" to leave this possibility open.

5. It would strengthen the data to knock out furin in oocytes or at least demonstrate furin.

R: We appreciate this concern. Our hypothesis relies on previously published data showing that the use of a furin inhibitor in oocytes prevents ENaC cleavage (7). This has also been shown in mammalian cells in culture with the furin inhibitor and by knocking out furin (6). Moreover, furin is a ubiquitous protein and it has been shown to be expressed in oocytes (13, 14) (https://www.xenbase.org/xenbase/static/gene/rna_seq_view.jsp).

6. Why was an accepted protease inhibitor not used as a positive control, i.e. aprotinin or alike to test that a similar inhibition as with S-protein overexpression was observed and to map the part of the reaction taking place on the cell surface.

R: We appreciate this comment. We are convinced that the observed fragments for αENaC and S protein are product of the previously described cleavage because: they have the expected sizes, the S fragment is not observed with the cleavage mutant, and the αENaC fragment is only observed in the presence of γENaC (as previously reported) (3). Thus, we did not think the protease inhibitor control to be necessary.

7. How do you compare densitometry across gels on the immunoblots – this should be described better/in detail.

R: We appreciate this comment. We have now included this information in the supplementary materials (within the Immunoblots section). The analysis was performed as follows. "For the analysis of samples across different gels, densitometries of each individual gel were first quantified, and the values of each sample were normalized to the average intensity of the samples within the gel. For the analysis of ENaC processing, values from the cleaved band (~65 kDa) were divided by the value of the uncleaved band (~90 kDa). The graph displays values relative to the control (ENaC without SPIKE). Group comparisons were performed using analysis of variance (ANOVA) , followed by Tukey post hoc test. A significance level of $p < 0.05$ was considered statistically significant (*), $p < 0.01$ (**) and $p < 0.001$ (***) were denoted for higher significance.

8. In figure 1, please state the expected migratory pattern of each protein species and how it compares with actual migration and place independent molecular weight markers. A C-terminal antibody against human αENaC would show proteins migrating putatively at 74, 51, and 48 kDa (non-glycosylated). Was that the case ?

R: We have now included all molecular weight markers in the figure. Also, in the figure legend we now include information on the expected migratory pattern. Alpha ENaC is cleaved in two positions by furin-like proteases that release a short inhibitory tract that is too small to be detected in the blots. Thus, with an antibody recognizing the C-terminus of the protein like the one we used (against the C-terminal HA tag) two bands are expected to be detected. In X. laevis oocytes (and also when overexpressed in mammalian cells), the bands observed are usually around the following weights: 90 kDa for the full length protein and a 65 kDa for the cleaved C-terminal fragment (3, 6, 15). We are aware that the predicted molecular weights for the full length protein and cleaved fragments are smaller, but it has been shown that ENaC bands are usually observed at higher molecular weights do to glycosylation (15).

9. Authors express alpha, beta and gamma ENaC -but I can only find immunoblotting data on alpha. Why are especially gamma ENaC not evaluated since it also depends on furin for cleavage and an extra cleavage to gain full activity in the intact channel.

R: Please refer to response to comment # 2 of reviewer #2.

10. Frankly, n=3 does not allow meaningful statistical evaluation (fig 1). Statistical methods are not described except for fig 2 data (or I cannot find it). Please state in figure legends.

R: We are sorry for the omission, and we have now included a description of the statistical methods used to analyze data on figure 1 in the supplemental information (Immunoblots section) and in the legend of the figure as requested by the reviewer. Given the observed robustness of the inhibition of αENAC cleavage observed and the relatively low variability of the data obtained, an n of 3 was sufficient to obtain statistically significant results.

11. What is "K18hACE2 transgenic mice" -please provide details. How many mice were used in total and how many died before inclusion- these parameters should be reported.

R: Thank you for this comment. We now provide details on K18hACE2 transgenic mice (on page 14 of the manuscript). These mice have been a widely used mouse model in SARS-CoV-2 research. We also provide the numbers of mice used.

"The k18 mouse is a frequently used mouse model in SARS-Cov-2 research that has been available since 2007 (16). It expresses the SARS-CoV-2 receptor (full length human angiotensin-converting enzyme 2 [hACE2]) essential for viral cell entry (17) under keratin 18 promoter which directs its expression to epithelia, including airway epithelia (16). This model develops a rapidly lethal infection after intranasal inoculation with the ancestral SARS-Cov-2 strain (18, 19)."

In addition, the Animals Experiments section of the manuscript was modified to mention this:

"All work with live SARS-CoV-2 was performed in the BSL-3 facility of the Ricketts Regional Biocontainment Laboratory, according to a protocol approved by the Institutional Animal Care and Use Committees of Northwestern University (approval number IS00004795) and University of Chicago (approval number 72642).
Ten K18hACE2 transgenic mice (5 male and 5 female) were inoculated with 2x104 PFU of SARS-CoV-2 (Washington strain) intranasally as previously reported by us (25). Animals were weighed once daily and monitored twice daily for health using a clinical scoring system (25). Animals that had lost >20% of their body weight or had a severely worsened clinical score (>3) were humanely euthanized which was

considered as fatal event. Based on the severity of clinical score, all mice had to be euthanized on days 6-7. The lung was taken from all animals and fixed in formalin. Formalin-fixed lungs were released from the BSL-3 facility after verifying absence of infectious virus and were then paraffin embedded and cut to slides (4 um) by the Mouse Histology and Phenotyping Laboratory Center, Northwestern University. We then performed lung tissue staining for ENaC from eight out of ten SARS-Cov-2-infected mice that had still enough tissue available for this study and compared it with the staining of lungs obtained from three uninfected control mice."

12. It is a pity that fresh lung tissue from the mice was not available for immunoblotting, however it would have some value to validate immunofluorescence by running immunoblots on non-infected control mice to demonstrate that the antigen is significantly present in the adult mouse lung. And compared to kidney.

R: It is well known that ENaC is expressed in lung at significant levels (20). The antibody we used has been successfully used in previous studies to detect gamma ENaC in immunoblots performed with lung samples (1). In our hands, the antibody also specifically recognized ENaC in kidney and lung tissue as suggested by the validation we performed. Please refer to response to comment 1 of reviewer #1 in which we explain this validation.

13. In contrast to the immunoblotting where only alpha ENaC data are shown, in immunofluorescence only gammaENaC is shown. Why? Please add data on alpha. In the legend to fig 3 it is stated "ENaC" but not alpha or gamma. Please clarify.

R: We were only able to analyze gamma ENaC with immunofluorescence since the commercially available alpha ENaC antibodies do not appear to specifically recognize the protein (1). In contrast, the available gamma ENaC antibody is quite good as shown by our validation data and by data from others (1). We are aware that it would have been ideal to analyze alpha ENaC. However, since there is evidence that absence of alpha ENaC impairs trafficking of beta and gamma ENaC to the plasma membrane (21) we were hoping to perhaps observe affected gamma ENaC expression or affected subcellular localization in type 2 pneumocytes of infected mice.

14. The number of the protocol approving the experiments and the date should be given.

R: We added the following sentence to the text of the manuscript that includes this information:

"All work with live SARS-CoV-2 was performed in the BSL-3 facility of the Ricketts Regional Biocontainment Laboratory, according to a protocol approved by the Institutional Animal Care and Use Committees of Northwestern University (approval number IS00004795) and University of Chicago (approval number 72642)."
We cannot provide a precise information on the date of approval, but since the protocol number is unique for animal studies performed at a given institution, we hope that the information provided would be satisfactory to the reviewer.

15. The magnificantion of the immunofluorescence and/or the resolution in my cope does not allow to see much detail. Could higher magnificantion be shown.

R: To comply with this Reviewer's suggestion and with the Reviewer 2 comment No. 5, we now provide insets of higher resolution in the new Figure 3.

16. Please delete the speculative parts of the discussion on dexamethasone, it is tangential, not tested in the study and although interesting, not relevant in the present context.

R: This has been removed as suggested by the reviewer.

References

1. **Mutchler SM**, **Shi S**, **Whelan SCM**, **Kleyman TR**. Validation of commercially available antibodies directed against subunits of the epithelial Na+ channel. *Physiol Rep* 11: 1–13, 2023. doi: 10.14814/phy2.15554.
2. **Grant SN**, **Lester HA**. Regulation of epithelial sodium channel activity by SARS-CoV-1 and SARS-CoV-2 proteins. *Biophys J* 120: 2805–2813, 2021. doi: 10.1016/j.bpj.2021.06.005.
3. **Harris M**, **Garcia-Caballero A**, **Stutts MJ**, **Firsov D**, **Rossier BC**. Preferential assembly of epithelial sodium channel (ENaC) subunits in Xenopus oocytes: Role of furin-mediated endogenous proteolysis. *J Biol Chem* 283: 7455–7463, 2008. doi: 10.1074/jbc.M707399200.
4. **Canessa CM**, **Schild L**, **Buell G**, **Thorens B**, **Gautschi I**, **Horisberger J**, **Rossier BC**. Amiloride-sensitive epithelial Na+ channel is made of three homologous subunits. *Nature* 367: 463–467, 1994. doi: 10.1038/367463a0.
5. **Romero MJ**, **Yue Q**, **Singla B**, **Hamacher J**, **Sridhar S**, **Moseley AS**, **Song C**, **Mraheil MA**, **Fischer B**, **Zeitlinger M**, **Chakraborty T**, **Fulton D**, **Gan L**, **Annex BH**, **Csanyi G**, **Eaton DC**, **Lucas R**. Direct endothelial ENaC activation mitigates vasculopathy induced by SARS-CoV2 spike protein. *Front Immunol* 14: 1–13, 2023. doi: 10.3389/fimmu.2023.1241448.
6. **Hughey RP**, **Bruns JB**, **Kinlough CL**, **Harkleroad KL**, **Tong Q**, **Carattino MD**, **Johnson JP**, **Stockand JD**, **Kleyman TR**. Epithelial Sodium Channels Are Activated by Furin-dependent Proteolysis. *J Biol Chem* 279: 18111–18114, 2004. doi: 10.1074/jbc.C400080200.
7. **Diakov A**, **Bera K**, **Mokrushina M**, **Krueger B**, **Korbmacher C**. Cleavage in the γ-subunit of the epithelial sodium channel (ENaC) plays an important role in the proteolytic activation of near-silent channels. *J Physiol* 586: 4587–4608, 2008. doi: 10.1113/jphysiol.2008.154435.
8. **Thomas G**. Furin at the cutting edge: From protein traffic to embryogenesis and disease. *Nat Rev Mol Cell Biol* 3: 753–766, 2002. doi: 10.1038/nrm934.
9. **Jean F**, **Thomas L**, **Molloy SS**, **Liu G**, **Jarvis MA**, **Nelson JA**, **Thomas G**. A protein-based therapeutic for human cytomegalovirus infection. *Proc Natl Acad Sci U S A* 97: 2864–2869, 2000. doi: 10.1073/pnas.050504297.
10. **Kashlan OB**, **Kleyman TR**. ENaC structure and function in the wake of a resolved structure of a family member. *Am J Physiol - Ren Physiol* 301, 2011. doi: 10.1152/ajprenal.00259.2011.
11. **Rossier BC**, **Jackson Stutts M**. Activation of the epithelial sodium channel (ENaC) by serine proteases. *Annu Rev Physiol* 71: 361–379, 2009. doi: 10.1146/annurev.physiol.010908.163108.
12. **Anand D**, **Hummler E**, **Rickman O**. ENaC activation by proteases. *Acta Physiol* : 1–13, 2022. doi: 10.1111/apha.13811.
13. **Nelsen S**, **Berg L**, **Wong C**, **Christian JL**. Proprotein convertase genes in Xenopus development. *Dev Dyn* 233: 1038–1044, 2005. doi: 10.1002/dvdy.20378.
14. **Session AM**, **Uno Y**, **Kwon T**, **Chapman JA**, **Toyoda A**, **Takahashi S**, **Fukui A**, **Hikosaka A**, **Suzuki A**, **Kondo M**, **Van Heeringen SJ**, **Quigley I**, **Heinz S**, **Ogino H**, **Ochi H**, **Hellsten U**, **Lyons JB**, **Simakov O**, **Putnam N**, **Stites J**, **Kuroki Y**, **Tanaka T**, **Michiue T**, **Watanabe M**, **Bogdanovic O**, **Lister R**, **Georgiou G**, **Paranjpe SS**, **Van Kruijsbergen I**, **Shu S**, **Carlson J**, **Kinoshita T**, **Ohta Y**, **Mawaribuchi S**, **Jenkins J**, **Grimwood J**, **Schmutz J**, **Mitros T**, **Mozaffari S V.**, **Suzuki Y**, **Haramoto**

Y, Yamamoto TS, Takagi C, Heald R, Miller K, Haudenschild C, Kitzman J, Nakayama T, Izutsu Y, Robert J, Fortriede J, Burns K, Lotay V, Karimi K, Yasuoka Y, Dichmann DS, Flajnik MF, Houston DW, Shendure J, Dupasquier L, Vize PD, Zorn AM, Ito M, Marcotte EM, Wallingford JB, Ito Y, Asashima M, Ueno N, Matsuda Y, Veenstra GJC, Fujiyama A, Harland RM, Taira M, Rokhsar DS. Genome evolution in the allotetraploid frog Xenopus laevis. *Nature* 538: 336–343, 2016. doi: 10.1038/nature19840.

15. Hughey RP, Mueller GM, Bruns JB, Kinlough CL, Poland PA, Harkleroad KL, Carattino MD, Kleyman TR. Maturation of the epithelial Na+ channel involves proteolytic processing of the ??- and ??-subunits. *J Biol Chem* 278: 37073–37082, 2003. doi: 10.1074/jbc.M307003200.

16. McCray PB, Pewe L, Wohlford-Lenane C, Hickey M, Manzel L, Shi L, Netland J, Jia HP, Halabi C, Sigmund CD, Meyerholz DK, Kirby P, Look DC, Perlman S. Lethal Infection of K18- hACE2 Mice Infected with Severe Acute Respiratory Syndrome Coronavirus . *J Virol* 81: 813–821, 2007. doi: 10.1128/jvi.02012-06.

17. Batlle D, Monteil V, Garreta E, Hassler L, Wysocki J, Chandar V, Schwartz RE, Mirazimi A, Montserrat N, Bader M, Penninger JM. Evidence in favor of the essentiality of human cell membrane-bound ACE2 and against soluble ACE2 for SARS-CoV-2 infectivity. *Cell* 185: 1837–1839, 2022. doi: 10.1016/j.cell.2022.05.004.

18. Hassler L, Wysocki J, Gelarden I, Sharma I, Tomatsidou A, Ye M, Gula H, Nicoleascu V, Randall G, Pshenychnyi S, Khurram N, Kanwar Y, Missiakas D, Henkin J, Yeldandi A, Batlle D. A Novel Soluble ACE2 Protein Provides Lung and Kidney Protection in Mice Susceptible to Lethal SARS-CoV-2 Infection. *J Am Soc Nephrol* 33: 1293–1307, 2022. doi: 10.1681/ASN.2021091209.

19. Hassler L, Wysocki J, Ahrendsen JT, Ye M, Gelarden I, Nicolaescu V, Tomatsidou A, Gula H, Cianfarini C, Forster P, Khurram N, Singer BD, Randall G, Missiakas D, Henkin J, Batlle D. Intranasal soluble ACE2 improves survival and prevents brain SARS-CoV-2 infection. *Life Sci alliance* 6, 2023. doi: 10.26508/lsa.202301969.

20. Matalon S, Bartoszewski R, Collawn JF. Role of epithelial sodium channels in the regulation of lung fluid homeostasis. *Am J Physiol - Lung Cell Mol Physiol* 309: L1229–L1238, 2015. doi: 10.1152/ajplung.00319.2015.

21. Rubera I, Loffing J, Palmer LG, Frindt G, Fowler-jaeger N, Sauter D, Carroll T, Mcmahon A, Hummler E, Rossier BC. Collecting duct–specific gene inactivation of αENaC in the mouse kidney does not impair sodium and potassium balance. *J Clin Invest* 112, 2003. doi: 10.1172/JCI200316956.Introduction.
